# Prostaglandin E_2_-EP2 signaling as a node of chronic inflammation in the colon tumor microenvironment

**DOI:** 10.1186/s41232-017-0036-7

**Published:** 2017-03-01

**Authors:** Tomohiro Aoki, Shuh Narumiya

**Affiliations:** Innovation Center for Immunoregulation Technologies and Therapeutics (AK Project), Kyoto University Graduate School of Medicine, Konoe-cho Yoshida, Sakyo-ku, Kyoto City, Kyoto 606-8501 Japan

**Keywords:** Prostaglandin, EP2, Inflammation, Microenvironment, Colon cancer, Neutrophil, Fibroblast, CXCL1, TNF-α, COX-2

## Abstract

**Background:**

Colorectal cancer is the third most common cancer. Involvement of prostaglandin (PG) system in the pathogenesis of colorectal cancer has been suggested from clinical studies demonstrating therapeutic effect of NSAIDs including aspirin or selective COX-2 inhibitors. However, mechanisms on how PG regulates inflammatory responses leading to colorectal cancer development remain obscure. Further, careful attention is needed to use these drugs for a long time because of adverse effects due to non-specific inhibition of physiological PG production in addition to pathological one, making the development of alternatives to aspirin important. Recent studies using mouse model of colitis-associated colon cancer, azoxymethane (AOM)-dextran sodium sulfate (DSS) model, have revealed some of the mechanisms on how PG regulates inflammation in lesions and proposed PG receptor as a therapeutic target.

**Main body of abstract:**

Among each PG receptor subtype examined, prostaglandin E receptor 2 (EP2) signaling specifically contributes to colorectal cancer formation and inflammation in lesions of AOM-DSS model. EP2 is expressed in neutrophils, infiltrated major inflammatory cells, and tumor-associated fibroblasts (TAFs) in the tumor stroma of this mouse model and also in clinical specimen from ulcerative colitis-associated colorectal cancer. Bone marrow transfer experiments between wild-type and EP2-deficient mice have confirmed the involvement of EP2 signaling in these two types of cells in the pathogenesis of the disease. EP2 signaling in both types of cells regulates the transition to and maintenance of inflammation in multiple steps to shape the tumor microenvironment which contributes to trigger and promote colorectal cancer. In this process, PGE_2_-EP2 signaling synergizes with TNF-α to amplify TNF-α-induced inflammatory responses, forms a positive feedback loop involving COX-2-PGE_2_-EP2 signaling to exacerbate PG-mediated inflammation once triggered, and alternates active cell populations participating in inflammation through forming self-amplification loop among neutrophils. Thus, EP2 signaling functions as a node of inflammatory responses in the tumor microenvironment. Based on such a notion, EP2 can become a strong candidate for therapeutic target of colorectal cancer treatment. Indeed, in AOM-DSS model, a selective EP2 antagonist, PF-04418948, potently suppresses colorectal tumor formation.

**Short conclusion:**

PGE_2_-EP2 signaling functions as a node of chronic inflammation which shapes the tumor microenvironment and thus is a strong candidate of target for the chemoprevention of colorectal cancer.

## Background

Prostaglandins (PGs) including PGD_2_, PGE_2_, PGF_2α_, PGI_2_, and thromboxane (TX) A_2_ are arachidonic acid metabolites formed by sequential actions of cyclooxygenase (COX) and respective synthases for each PG and exert their actions by acting on their cognate G-protein-coupled receptors (GPCRs) [1]. PGs are traditionally recognized as a major mediator of acute inflammatory responses because non-steroidal anti-inflammatory drugs (NSAIDs), which inhibit the activity of COX and shut off PG production, effectively suppress symptoms of acute inflammation: fever, reddish, swelling, and pain. Interestingly, recent experimental studies mainly using mice deficient in each PG receptor subtype subjected to animal disease models have revealed the involvement of PG system and its receptor signaling in the pathogenesis of various diseases with chronic course such as cancer, vascular diseases, or neurodegenerative diseases and thereby suggested the regulation of not only acute inflammation but also chronicity of inflammation by PGs [2].

Colorectal cancer is the third common cancer [3]. One of the major risk factors of colorectal cancer is inflammatory bowel diseases such as ulcerative colitis [4], indicating the involvement of inflammatory responses in the pathogenesis of colorectal cancer. Indeed, in 1988, reduction of the risk of colorectal cancer development in aspirin users was reported [5]. Subsequently, some clinical studies reported reduction of the risk and mortality of colorectal cancer by the use of NSAIDs including aspirin [6–8], suggesting the close association of the pathogenesis of colorectal cancer with PG-mediated inflammation. Up to now, contribution of PG system to colon cancer cells has been extensively studied mainly using cancer cell lines, i.e., prostaglandin E receptor 2 (EP2) signaling promotes growth of colon cancer cells via driving a Gs-axin-b-catenin axis in vitro [9]. Although inflammation in the tumor microenvironment, where many types of cells interact with tumor cells, is essential to promote their development and growth, studies addressing how PG system regulates this inflammation in the tumor microenvironment of colorectal cancer in detail are quite limited [10, 11].

In this short review, we explain and interpret our recent experimental findings regarding the regulation of chronic inflammatory responses in the tumor microenvironment of colorectal cancer by PGE_2_-EP2 signaling cascade [12].

### Prostaglandin system as a node of inflammation in tumor environment and its contribution to colon tumor formation

To analyze the contribution of PG system to inflammatory responses in the tumor microenvironment and subsequent colon tumor formation, we used colitis-associated colorectal cancer model of mice, in which tumor is induced in the colon by the combination of administration of carcinogen, azoxymethane (AOM), with dextran sodium sulfate (DSS) to induce colitis [13]. Among the PG receptor subtypes examined, genetic deletion of EP2 (*Ptger2*) specifically and almost completely suppressed colon tumor formation in AOM-DSS model of mice [12]. Intriguingly, deletion of EP1 (*Ptger1*), EP3 (*Ptger3*), or some other PG receptor subtypes failed to suppress colon tumor formation but rather exacerbated it, suggesting the presence of PG receptor subtype oppositely functioning to colon tumor formation. Consistently, genetic deletion of mPGES-1 (*Ptges*), an enzyme producing PGE_2_, remarkably suppressed colon tumor formation, but the number of tumors induced in this mouse line was still significantly larger than that in EP2-deficient mice [12]. These experimental findings suggest the superiority of antagonism-targeting EP2 over non-specific inhibition of PG receptors through suppression of PGE_2_ production by NSAIDs or selective COX-2 inhibitors. Histopathological analyses revealed the remarkable reduction of inflammatory infiltrates accompanied with relatively conserved crypts in the colon of EP2-deficient mice. The majority of these inflammatory infiltrates is neutrophil, defined as a CD45^+^CD11b^+^Ly-6G^+^ cell, and deficiency of EP2 significantly decreased its number in the colon [12], suggesting the role of EP2 signaling in neutrophil infiltration into the colon and regulation of neutrophil-mediated inflammatory responses in lesions. In immunohistochemical analysis, EP2 expression was detected in these neutrophils and also in tumor-associated fibroblasts (TAFs) in mesenchyme [12]. In human specimen from ulcerative colitis-associated colorectal cancer, EP2 expression could be detected similarly in neutrophils and TAFs, suggesting the clinical relevance. In accordance with the expression of EP2 on neutrophils and TAFs, bone marrow transfer experiments between wild-type and EP2-deficient mice revealed the significant contribution of EP2 expressed on both recipient and donor cells [12]. In both cell types, EP2 signaling plays a role as a regulator of inflammatory responses. First, in in vitro study, EP2 signaling synergizes with other cytokines present in the tumor microenvironment, TNF-α, to transcriptionally induce expressions of IL-6 (*Il6*), COX-2 (*Ptgs2*), and CXCL1 (*Cxcl1*) in primary culture of neutrophils and IL-6, COX-2, and MMP-12 (*MMP12*) in 18 Co cell line, an in vitro TAF model [12]. Intriguingly, EP2 signaling alone only slightly induces these expressions. The in vivo relevance of these findings also demonstrated that deficiency in EP2 suppresses expressions of pro-inflammatory molecules, such as IL-6 and TNF-α, and factors supporting tumor cell growth, such as osteopontin, in lesions [12]. In other words, EP2 signaling amplifies TNF-α-induced expressions of pro-inflammatory factors like IL-6 as a “cytokine amplifier” [12]. Second, as described above, EP2 signaling induces COX-2 expression both in neutrophils and 18 Co cells synergistically with TNF-α in vitro [2, 12]. Consistently, because expressions of both EP2 and COX-2 are induced and become detectable in neutrophils and TAFs in the colon of AOM-DSS model during the development of the disease and genetic deletion of EP2 greatly suppressed COX-2 expression in these two types of cells [12], EP2 and COX-2 presumably have inter-dependency in vivo. As COX-2 is an inducible form of a PG-producing enzyme, induction of this enzyme under EP2 signaling means the formation of a positive feedback loop including COX-2-PGE_2_-EP2 signaling cascade. Such a formation of a positive feedback loop makes PG-mediated inflammatory responses once triggered long-lasting and being exacerbated. Third, in primary culture of neutrophils, EP2 signaling induced their own chemoattractant, CXCL1, synergistically with TNF-α [12]. In the colon of AOM-DSS model, CXCL1 expression was mainly detectable in infiltrated neutrophils and its expression was remarkably and significantly ameliorated in EP2-deficient mice [12], suggesting the regulation of CXCL1 expression by EP2 signaling also in vivo as in in vitro. EP2-mediated CXCL1 expression alternates active cell populations participating in inflammatory responses in situ via infiltrating neutrophils and thereby contributing to the configuration of the microenvironment where inflammation triggers/promotes tumorigenesis. Intriguingly, CXCL1 is produced from neutrophils both in in vitro and in vivo, suggesting the formation of a self-amplification loop among neutrophils which further exacerbates and prolongs inflammation.

**Fig. 1:**
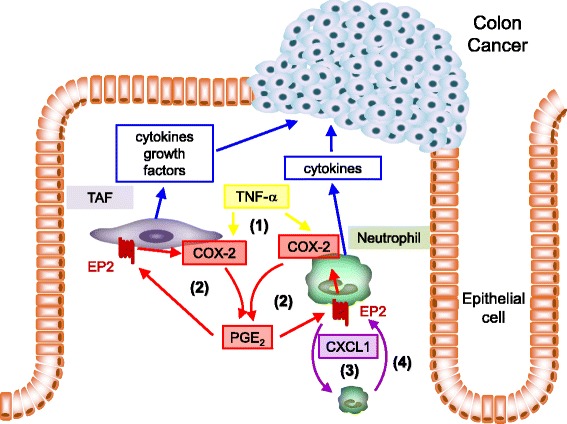
Regulation of inflammatory responses in the tumor microenvironment of colon cancer by PGE_2_-EP2 signaling. PGE_2_-EP2 signaling shapes tumor environment in multiple steps, i.e., (1) the synergistic induction of cytokines and growth factors from tumor-associated fibroblasts (TAFs) and neutrophils with TNF-α, (2) the formation of a positive feedback loop involving COX-2-PGE_2_-EP2 signaling to exacerbate inflammation once triggered, (3) the induction of CXCL1 to recruit neutrophils and alternate active cell populations in situ, and (4) the formation of a self-amplification loop among neutrophils

In summary, PGE_2_-EP2 signaling plays a role as a node of inflammation in the tumor microenvironment to amplify inflammatory responses which support tumor development and progression. In this process, this EP2 signaling contributes to the chronicity of inflammation in multiple steps (Fig. 1), i.e., (1) the formation of a positive feedback loop to exacerbate PG-mediated inflammatory responses, (2) the function as a “cytokine amplifier,” and (3) the alternation of active cell populations in situ partially through forming a self-amplification loop among neutrophils. Such a role of EP2 or PG receptor signaling on chronicity of inflammation can be observed also in the microenvironment of various inflammatory settings, and thus, PG system contributes to the pathogenesis of various diseases as a node of inflammatory responses. Although such roles of PGs should be verified in more detail, understanding of PG system as a major regulator of inflammation in not only acute but also chronic one is conceptually important for our understanding of the pathogenesis underlying various diseases and also as a translational research to develop novel therapeutic drugs targeting inflammatory diseases.

### Potential of EP2 as a therapeutic target to treat colorectal cancer

As described above, EP2 signaling plays a crucial role in colon tumorigenesis as a node of inflammation in the tumor microenvironment. Thereby, EP2 may be a strong candidate as a therapeutic target for the treatment of colorectal cancer especially with active inflammatory responses in the stroma. The potential of pharmacological inhibition of EP2 signaling as a therapeutic measure for colorectal cancer treatment is therefore examined in AOM-DSS model of mice [12]. A selective EP2 antagonist, PF-04418948 [14, 15], is administered to mice subjected to AOM-DSS model, and colon tumor formation in this model is then examined. As a result, PF-04418948 dose dependently (1–100 mg/kg) suppresses the number of colon tumor, and notably at the highest dose of this compound (100 mg/kg), any colon tumor is induced [12]. Consistent with the EP2-mediated formation of a self-amplification loop among neutrophils via secretion of their chemoattractant CXCL1, infiltration of inflammatory cells and expression of CXCL1 in the lesion are both remarkably suppressed in PF-04418948-treated mice [12]. A selective EP2 antagonist can therefore become a strong candidate for drugs to treat colorectal cancer or prevent the initiation/recurrence of it via acting on inflammatory responses in situ. Here, it should be carefully discussed that NSAIDs and COX-2 inhibitors have significant adverse effects such as gastrointestinal hemorrhage and cardiovascular accidents [16] derived from a non-specific inhibition of PG receptors, some of which mediates a physiological function to maintain homeostasis of organs, and impaired balance between PGI_2_ and TXA_2_, respectively. Indeed, deficiency in COX-1 or COX-2 potentiates injury in the colon epithelium under treatment with DSS in mice [17]. Also, other anti-inflammatory drugs such as those targeting IL-6 and TNF-α have a potential risk of development of infectious and other types of cancers. Considered with normal development of EP2-deficient mice except for impaired fertilization [18] and the induced expression of EP2 selectively in lesions, a selective EP2 antagonist can be a safer and potent alternative to aspirin and other anti-inflammatory drugs in the chemoprevention of colorectal cancer. Further, because the use of aspirin reduces the risk of death from all cancers including colorectal cancer, lung cancer, and esophageal cancer [19] and high expression of EP2 in tumor lesions have a positive correlation with poor outcome in some cancers [20, 21], a selective EP2 antagonist can also be a strong candidate as a therapeutic drug to treat a variety of cancers.

## Conclusions

Colorectal cancer is considered as a PG-mediated pathology through various clinical studies which have demonstrated the therapeutic effect of NSAIDs or selective COX-2 inhibitors on this disease. We addressed mechanisms on how PG system regulates colon tumorigenesis, and our recent experimental findings using AOM-DSS model in mice have demonstrated the involvement of PGE_2_-EP2 system in the pathogenesis of colorectal cancer. EP2 expresses in infiltrated neutrophils and TAFs in the tumor microenvironment, and this signaling plays a role to maintain inflammatory responses in the colon to form a microenvironment supporting the initiation and promotion of cancer cells. The processes EP2 signaling regulates such as the maintenance of inflammation include the formation of a positive feedback loop to amplify PG-mediated inflammation, the amplification of inflammation synergistically with other cytokines, and the alternation of cell populations participating in situ. Further, we propose the potential of a selective EP2 agonist as a safer alternative to aspirin.

## Abbreviations

AOM: Azoxymethane
DSS: Dextran sodium sulfate
EP2: Prostaglandin E receptor 2
PG: Prostaglandin
COX: Cyclooxygenase
